# A Study on the Impact of Fiscal Decentralization on Green Development from the Perspective of Government Environmental Preferences

**DOI:** 10.3390/ijerph19169964

**Published:** 2022-08-12

**Authors:** Ruichao Liu, Xiaoyan Zhang, Pengcheng Wang

**Affiliations:** 1 School of Economics, Qingdao University, Qingdao 260071, China; 2 College of Finance and Economics, Shandong University of Science and Technology, Taian 271000, China

**Keywords:** fiscal decentralization, green development, government environmental preferences, moderating effects

## Abstract

Green development is necessary for China to carry out high-quality economic development. As an important institutional arrangement in the vertical government structure, fiscal decentralization supports regional green development. Local government environmental preferences indicate local environmental protection awareness and affect the process of regional green development to a certain extent. Based on the review of relevant theories and literature, this study conducts an empirical analysis based on Chinese provincial panel data from 2007 to 2019 using a two-way fixed effects model and a panel threshold model. Both revenue decentralization and expenditure decentralization have a U-shaped nonlinear relationship with the green development efficiency calculated by the superefficiency SBM model, which includes undesirable output. Neither factor has a positive effect on green development efficiency at this stage, while local government environmental preferences are positively connected to green development efficiency. Furthermore, a moderating effect is observed in the relationship between fiscal decentralization and green development efficiency. Under the same level of revenue decentralization or expenditure decentralization, the stronger the environmental preferences of the local authority are, the higher the green development efficiency. This moderating effect is more significant in coastal areas than inland areas. Further research reveals a threshold for the moderating effect of local government environmental preferences on fiscal decentralization on green development. When the environmental preferences of local government are below the threshold, both revenue decentralization and expenditure decentralization significantly inhibit the improvement in green development efficiency. After the threshold is passed, the negative effects of both on green development efficiency are markedly curtailed. Then, the government performance appraisal system should be further optimized, fiscal decentralization reform should be strengthened, local financial investment in environmental protection should be expanded, and synergistic regional development should be promoted. China’s green development should be promoted to an advanced stage.

## 1. Introduction

The rapid expansion of human activity is driving environmental change. All indicators of Earth’s vitality continue to decline emphatically. Current development patterns weaken the Earth’s limited capacity to sustain human well-being. The world needs fundamental changes in society, the economy, and life. When calculating economic performance, it is important to focus on nature in addition to gross domestic product. In pursuing wealth and security, humanity must learn to value “natural capital”.

China’s economy relied on “factor input-driven” to create the “China Miracle” of sustained high-speed growth, but the tightening of resource constraints and the bottoming out of the ecological red line have become the shackles of development. To effectively solve the environmental problems accompanying economic development, since the concept of green development was put forward in 2015, the Chinese government has repeatedly stressed the importance of focusing on promoting the construction of material civilization and ecological civilization. “Promoting green development” and “green becoming the universal form” are important themes of China’s economic and social development in the present.

Fiscal decentralization is an important institutional arrangement in the vertical government structure. It greatly affects the method of regional economic development. Relying on local information advantages and cost advantages, fiscal decentralization can enhance the efficiency of resource allocation within a region and thus can promote regional green development. However, it can also motivate local governments to pursue economic growth, motivating them to distribute more resources to economic construction sectors and crowd out inputs in sectors with low economic benefits, such as environmental management.

With the deepening of China’s market economic system reform, the market plays a decisive role in resource allocation, but this does not mean that all projects can be implemented in any region. Local governments are responsible for the affairs of their jurisdiction, such as “three lines and one order” (ecological protection red line, environmental quality bottom line, resource utilization line, and ecological environment access list), which clarify differentiated restrictions for specific projects and industries and requirements for reducing production capacity in different regions. This means that government behavioral preferences directly influence the direction of regional development, having an important impact on the role of fiscal decentralization. It is crucial for promoting the development of regional green transformation.

Improving the efficiency of green development is the core of promoting green modernization, and rationalizing the financial relationship between the central and local governments is an inherent need to implement “green has become a common form”. As a reflection of their environmental awareness, local government environmental preferences can effectively regulate the relationship between fiscal decentralization and green development. Therefore, it is necessary to integrate fiscal decentralization, local government environmental preferences, and green development into a unified research framework and explore the effect of fiscal decentralization on the level of green development and how local government environmental preferences affect their relationship, explaining the causes of fluctuations in green development efficiency. Compared with many existing studies, this paper makes the following possible contributions.

(1) This paper complements and tests the basic theory of green development. Regional development is a multidimensional integration of the economy, environment, etc. This paper breaks away from the obvious object bias in previous studies. The concept of green development efficiency is used to incorporate economic growth and environmental pollution into the same analytical framework. On this basis, this paper introduces fiscal decentralization into the above framework and explores the impact effects of fiscal decentralization more comprehensively. It also strengthens the correlation between the economic impact and the environmental impact of fiscal decentralization. Furthermore, it expands the theoretical basis and impact effects of fiscal decentralization to promote the realization path of green development.
(2) The study has important empirical implications for understanding the differences in green development levels within developing countries. Considering the dual attributes and functions of Chinese local governments in charge of jurisdictional affairs, this paper further explores how the behavioral choice preferences of local governments affect the role of fiscal decentralization, incorporating local government environmental preferences into the framework. The paper tests the compensating effect of proactive local government actions in the environmental protection field in the context of fiscal decentralization to promote green development. It argues for the effectiveness of the current policy system on influencing local government incentives and constraints based on appropriate extensions.

The rest of this paper is organized as follows. Section 2 is the literature review, Section 3 is the empirical design, Section 4 is the empirical analysis, Section 5 is the research conclusion, and Section 6 is the policy recommendations.

## 2. Literature Review

### 2.1. A Study on the Measurement and Influencing Factors of Green Development

Two main methods are used to measure the level of green development: indicator evaluation and the efficiency measurement method. The representative system is the green development evaluation system proposed by the Organization for Economic Cooperation and Development (OECD), the UN Environment Programme (UNEP), etc. China has also achieved relatively fruitful results in this area [1]. Green development efficiency can fully consider the cost of energy consumption and environmental pollution in the process of economic development. Data envelopment analysis (DEA) efficiency measurement can prevent the strong assumption bias of stochastic frontier analysis. It better applies to multi-input, multioutput production activities. Many scholars use the SBM model with undesired outputs and its superefficiency model to carry out the measurement [2,3].

Scholars have also conducted a series of studies on the factors affecting green development. Foreign direct investment facilitates the diffusion and exchange of advanced technologies. It can promote green and sustainable development in inflowing regions. Among them, green investment has a positive impact on reducing environmental risks and achieving low-carbon green growth [4]. Both generic technological innovation and specific green technological capabilities contribute to the level of green development. They are the core elements for achieving green growth [5]. It has also been shown that some factors positively impact the improvement in green development, such as environmental decentralization [6], financial agglomeration [7], and advanced and rationalized industrial structure [8]. However, competition among local governments [9] and vertical fiscal imbalance [10] is not conducive to green development in the region.

### 2.2. A Study of the Impact of Fiscal Decentralization on Economic Growth and Environmental Quality

There are relatively few studies that directly consider the relationship between fiscal decentralization and green development. The impact of fiscal decentralization is mainly explored considering two subsystems: economic growth and environmental quality.

Previous studies have generally concluded that fiscal decentralization provides incentives for local policies that are conducive to economic growth. It can also increase long-term economic growth rates [11]. The above proposition is supported by single-country and cross-country empirical studies [12,13]. Fiscal decentralization stimulates local governments to provide more and better public goods and services. It can effectively solve the problem of heterogeneity of environmental preferences and trigger the phenomenon of environmental “competition for the upstream”. This is conducive to the improvement in environmental standards [14,15,16]. Ji et al. (2020) state that fiscal decentralization reduces CO_2_ emissions and significantly improves environmental quality [17]. However, a nonlinear relationship between fiscal decentralization and the two has also been proposed [18,19]. Fiscal decentralization does not always promote economic growth; it can even have a negative impact on economic growth [20]. Local public goods, such as the environment, can also be undersupplied. This is coupled with local governments’ free-riding behavior. Both are detrimental to environmental pollution control and hinder the improvement in environmental quality [21].

Several empirical studies at the provincial and city levels agree that fiscal decentralization is a key factor in China’s rapid economic growth and may be the greatest contributor [22]. A nonlinear effect of fiscal decentralization on economic growth is also shown [23]. Empirical studies at different levels also emphasize that fiscal decentralization increases environmental pollution in the local and surrounding regions. This is detrimental to the improvement in environmental quality [24]. This effect may decrease and then increase as the level of economic development increases. It may also be reversed by an increase in the degree of fiscal decentralization [25,26].

### 2.3. A Study on the Behavioral Preferences of Local Government

There are relatively few specific research results on the environmental preferences of local governments. Scholars mainly focus on the relationship between local government environmental preferences and environmental quality.

The central government prefers society-wide welfare maximization. However, local governments have relatively more short-term goals, while local government preferences are not always aligned with the central government. To achieve the goal of maximizing benefits, local governments adjust their behavioral preferences. This adjustment is based on changes in promotion incentives and regional realities [27]. Multi-objective decision making prefers capital investment in quick results and significant economic growth effects. The supply of public goods such as the environment, which has no significant short-term economic effects, is neglected [28]. An increase in local government environmental preferences can significantly enhance the environmental governance effect. It can also improve total social welfare to some extent [29].

From previous studies, fiscal decentralization has an important impact on regional economic growth and environmental quality. However, little literature analyzes both the economy and the environment in an integrated manner; to a certain extent, it deviates from the era meaning of China’s green development. Therefore, there is no agreement on the combined effect of fiscal decentralization on green development in China. Moreover, established studies have also downplayed the role of local government behavioral preferences in these effects, reducing the guiding significance of the conclusion to the development of green modernization. Based on the concept of green development efficiency, this paper incorporates the multidimensional impacts of fiscal decentralization and local government environmental preferences on economic growth and environmental quality in China into the same research system. This also adds to the deficiencies in the current study.

## 3. Empirical Design

### 3.1. Research Methods and Model Design

#### 3.1.1. Green Development Efficiency Measurement Model

The Super-SBM model includes undesired outputs and can effectively solve the problem of input and output slackness. It can also account for the undesired outputs, such as pollutants, generated in the production process. It can better solve the problem of ranking effective DMUs [30]. The measurement results from VRS conditions are more realistic [31]. Therefore, this model is chosen to measure green development efficiency (*GDE*) in this paper, and the model is represented as follows:

(1) ρ=min[1+1m∑i=1msi−xik1−1q1+q2(∑r=1q1sr+yrk+∑t=1q2stb−btk)]s.t.∑j=1,j≠knxijλj−si−≤xik∑j=1,j≠knyrjλj+sr+≥yrk∑j=1,j≠knbtjλj−stb−≤btk1−1q1+q2(∑r=1q1sr+yrk+∑t=1q2stb−btk)>0λ,s−,s+≥0i=1,2,⋯,m;r=1,2,⋯,q1;t=1,2,⋯,q2;j=1,2,⋯,n(j≠k)

where *ρ* represents the efficiency value of the decision unit, which is greater than 0 or greater than 1. The larger the value is, the higher the efficiency of the decision unit. *x_ik_*, *y_rk_*, and *b_tk_* represent the input, desired output, and undesired output, respectively. *m*, *q*_1_, and *q_2_* represent the number of inputs, desired outputs, and undesired outputs, respectively. *s_i_^−^*, *s_r_^+^*, and *s_t_^b^*^−^ represent the slack variables of input, desired output, and undesired output, respectively. When the slack variables are greater than 0, there is room for improvement in the efficiency value of the decision unit. n is each decision unit; *λ_j_* represents the weight.

#### 3.1.2. Modeling the Effect of Fiscal Decentralization on the Efficiency of Green Development

The emergence of fiscal decentralization is intended to motivate local governments to take part in social management. They are free to choose the type of policy that suits their actual situation. Thus, they can better supply public services to local residents and achieve the highest regional social welfare.

Local governments have a certain degree of autonomy under fiscal decentralization. This is conducive to local governments’ better use of their information and cost advantages. They can flexibly allocate resources, adjust policies according to the specific conditions within the region, and coordinate the relationship between economic development and environmental protection. Fiscal decentralization can also improve the efficiency of factor utilization and support the effective management of environmental problems. Ultimately, it promotes the realization of regional green development goals. However, in the presence of self-interest motives [32] and the mismatch of financial and administrative powers [33], fiscal decentralization motivates local governments to promote rapid economic growth as their first priority. However, it belittles the management of environmental problems and may even lower the environmental access threshold to introduce more enterprises into the jurisdiction. This is detrimental to the harmonious development of the economy and the environment and has a negative effect on improving the level of green development in the region.

In summary, it can be seen that the direction of the impact of fiscal decentralization on green development is uncertain. Moreover, the existence of nonlinear effects is not excluded. To study the effect of fiscal decentralization on the efficiency of green development, a two-way fixed-effect model (2) is established.

(2) $$GDE_{it}=\alpha_{0}+\alpha_{1}FD_{it}+\alpha_{2}FD_{it}^{2}+\alpha_{3}Control_{it}+\mu_{i}+\eta_{t}+\varepsilon_{it}$$

where *GDE* represents the explanatory variable, which is the green development efficiency value. *FD* represents the degree of fiscal decentralization, while the quadratic term *FD*^2^ of fiscal decentralization is added to the model to examine whether there is a nonlinear relationship between fiscal decentralization and green development efficiency. Control represents a series of control variables affecting green development efficiency. *μ* is the region fixed effect. *η* is the time fixed effect. ε represents the disturbance term. *i* represents the region. *t* represents the year. If α_1_ is significant and α_2_ is insignificant, fiscal decentralization has a linear effect on green development efficiency. If α_2_ is significant, there is a nonlinear relationship between fiscal decentralization and green development efficiency.

#### 3.1.3. A Model of the Moderating Effect of Local Government Environmental Preferences on Fiscal Decentralization Affecting Green Development Efficiency

This paper defines local government environmental preferences as the degree of importance local governments attach to regional environmental quality. It reflects the strength of the local government’s will to govern the environment and is an important reflection of local environmental protection awareness. The impact of fiscal decentralization on green development is closely linked with local government environmental preferences.

Faced with increasing pressure from the central government’s environmental policy, the local government’s motivation to manage the environment is greatly enhanced, and preferences for environmental quality are strengthened. Enhanced environmental preferences of the local government can lead to more funds being used for environmental management. It can solve all kinds of problems, such as environmental pollution and ecological damage. It can also guide the flow of resources in the region to the green environmental protection field. This helps build a good green industrial system and determine new growth points in economic development. In turn, it can better balance economic growth and environmental protection and promote the region to achieve a higher level of green development.

In summary, the local government can always uphold the concept of green development. If the local government can always uphold the concept of green development, it can increase its preferences for regional environmental quality and insist on real environmental protection. Then, with the same degree of fiscal decentralization, the level of green development is higher. Referring to the practice of related scholars [34,35], the study establishes model (3) to examine the moderating effect of local government environmental preferences on the influence of fiscal decentralization on green development efficiency [34,35].

(3) $$\begin{array}{ll} GDE_{it}= & \beta_{0}+\beta_{1}FD_{it}+\beta_{2}FD_{it}^{2}+\beta_{3}Pre_{it}+\beta_{4}FD_{it}\times Pre_{it}\\ & +\beta_{5}Control_{it}+\mu_{i}+\eta_{t}+\varepsilon_{it} \end{array}$$

The above model adds the moderating variable local government environmental preferences and the interaction term between fiscal decentralization and local government environmental preferences based on model (2), where *Pre* denotes local government environmental preferences and *fd × Pre* denotes the interaction term between local government environmental preferences and fiscal decentralization. The meanings of the remaining symbols are consistent with those of model (2). If the coefficient of the interaction term *β*_4_ is significant, then local government environmental preferences moderate the relationship between fiscal decentralization and green development efficiency.

#### 3.1.4. A Spatial Effect Model of Local Government Environmental Preferences Regulating the Impact of Fiscal Decentralization on Green Development

Based on model (3), the study adds the spatial lag term of the explanatory variable green development efficiency for regression analysis. This is used to further consider the spillover effect of green development efficiency between regions. Based on the results of the Hausman test and the joint significance test (LR test), a time-individual double fixed effect model (4) is established.

(4) $$\begin{array}{ll} GDE_{it}= & \rho WGDE_{it}+\gamma_{0}+\gamma_{1}FD_{it}+\gamma_{2}FD_{it}^{2}+\beta_{3}Pre_{it}\\ & +\gamma_{4}FD_{it}\times Pre_{it}+\gamma_{5}Control_{it}+\mu_{i}+\eta_{t}+\varepsilon_{it} \end{array}$$

where *ρ* is the spatial autoregressive coefficient and *W* is the spatial weight matrix. The spatial weight matrix must be selected before conducting the spatial correlation test. The study reports the estimation results of the three commonly used spatial matrices: the spatial adjacency matrix *W*_1_, the geographic distance matrix *W*_2_ and the economic distance matrix *W*_3_ (Table 1).

#### 3.1.5. A Threshold Effect Model of Local Government Environmental Preferences Regulating the Impact of Fiscal Decentralization on Green Development

The study establishes a panel threshold model (5) with local government environmental preferences as the threshold variable to explore the effect of fiscal decentralization on green development under different degrees of local government environmental preference.

(5) $$\begin{array}{ll} GDE_{it}= & \theta_{0}+\theta_{1}FD_{it}I\left(pre_{it}\leq \psi \right)+\theta_{2}FD_{it}I(pre_{it}\geq \psi )\\ & +\theta_{3}Control_{it}+\mu_{i}+\eta_{t}+\varepsilon_{it} \end{array}$$

Since the number of thresholds needs to be estimated before it can be determined, a single threshold model is set first, where *I*(·) is the indicative function, *ψ* is the threshold value to be estimated, and the rest of the symbolic meanings are consistent with those in the baseline model.

### 3.2. Variable Description and Data Sources

#### 3.2.1. Variable Description

(1) Explained Variables

Green development requires both “green” and “development”. In economic development, the cost of resources and the environment must be given attention, with adherence to the priority of ecological benefits and taking resources and the ecological environment as hard constraints. The goals are to maximize the resource utilization rate, reduce pollution emissions and maximize economic benefits with the lowest ecological costs.

In this paper, green development efficiency (*GDE*) is defined as the ability to simultaneously reduce resource consumption and pollution emissions from undesired outputs while increasing desired outputs. This is the combined economic efficiency after considering resource costs and environmental costs. It offers a more comprehensive measure of a region’s green development level.

To ensure the scientificity and rationality of the selected input indicators, after a comprehensive review of the relevant literature, four indicators, namely, capital input, labor input, energy input and land input, are selected in this paper. The selected output indicators include two parts: desired output and undesired output. Desired output is characterized by gross regional product (*GDP*). The undesired output consists of carbon dioxide emissions, sulfur dioxide emissions, chemical oxygen demand emissions, and general industrial solid waste generation.

(2) Core Explanatory Variables

Fiscal decentralization (*FD*). According to the different perspectives examined, fiscal decentralization can be divided into two categories. One is revenue decentralization (*FRD*), which reflects the fiscal revenue power of the central government and local government. The other is expenditure decentralization (*FED*), which reflects fiscal expenditure power between the central and local governments. The following formula is used for the measurement of fiscal decentralization indicators:

Revenue decentralization (*FRD1*) = fiscal revenue per capita in the budget of each province/fiscal revenue per capita in the budget of the country.

Expenditure decentralization (*FED1*) = fiscal expenditure per capita in the provincial budgets/fiscal expenditure per capita in the national budget.

In addition, the second set of core explanatory variables indicators FRD2 and FED2 are selected for robustness testing in this paper.

Revenue decentralization (*FRD2*) = fiscal revenue per capita in each province’s budget/fiscal revenue per capita in the central budget.

Expenditure decentralization (*FED2*) = fiscal expenditure per capita in each province’s budget/fiscal expenditure per capita in the central budget.

The effect of fiscal decentralization on the efficiency of green development is uncertain, and the presence of nonlinear effects cannot be excluded. Therefore, the sign of the estimated coefficients is uncertain.

(3) Moderator Variables

Local government environmental preferences (*Pre*) are chosen as the regulating variable. Local government environmental preferences reflect the degree of importance the local government places on environmental quality. A government with a long-term perspective will consider the intertemporal positive externalities of environmental management and impose stricter environmental controls. Government environmental spending can be seen as an act of environmental regulation by the government and can reflect the government’s will to carry out environmental governance. Therefore, the share of energy conservation and environmental protection expenditures in general public budget expenditures is used to measure local government environmental preferences. A higher share indicates a deeper preference of local government for environmental quality and a higher intensity of environmental regulation. Local government environmental preferences are conducive to the improvement in green development efficiency. Therefore, the sign of its estimated coefficient is expected to be positive.

(4) Control Variables

① The first control variable is the level of economic development (*Pgdp*), which is measured by regional per capita gross domestic product. As the level of economic development continues to increase, people’s awareness of environmental protection also increases, and they pay more attention to the quality of the environment. Therefore, it is expected that the level of economic development will positively impact the efficiency of green development, and the sign of the estimated coefficient will be positive.

② The second control variable is trade openness (*Ope*). It is measured by the total import and export trade ratio and regional GDP of each region. On the one hand, expanding the degree of trade openness is conducive to the technology spillover effect, which enables a region to absorb more advanced green technologies and promotes improvement in the regional green development level. On the other hand, in China’s export trade, the proportion of high energy-consuming and heavy pollution products is quite high. The increase in trade openness leads to excessive consumption of resources and serious pollution emissions. Accordingly, the impact of trade openness on the efficiency of green development is uncertain.

③ The third control variable is advanced industrial structure (*Ind*). The advanced industrial structure reflects the transformation from secondary industry to tertiary industry. The increase in the advanced degree is conducive to the reduction in energy consumption and pollution emissions. It is measured by the ratio of the value added of the tertiary industry to the value-added of the secondary industry. Therefore, an advanced industrial structure is expected to be positively related to green development efficiency, with a positive sign of the estimated coefficient.

④ The fourth control variable is foreign direct investment (*Pfdi*). This is measured by the actual amount of FDI utilized per capita. According to the “pollution haven” hypothesis, developed countries transfer polluting enterprises to developing countries with a relatively low intensity of environmental regulations. This increases the level of environmental pollution in the host country. On the other hand, the introduction of foreign direct investment can bring advanced technologies, such as green technology, to the host country and produce a demonstration effect. This is conducive to the improvement in the green development level. Therefore, the impact of FDI on the efficiency of green development is uncertain.

⑤ The fifth control variable is research and development intensity (*RD*). It is measured by the ratio of internal expenditure on research and experimental development to regional GDP. R&D is an important source of technological progress, and increasing investment in R&D can effectively improve the level of scientific and technological development in the region. This improves the output capacity of enterprises and changes the situation of “high input, high pollution and low output” due to backward production methods. It also helps provide more effective pollution control technologies. Therefore, R&D intensity is expected to be positively related to green development efficiency, and the estimated coefficient sign is positive.

#### 3.2.2. Data Sources

The data used in this paper are for 30 provincial-level administrative regions of China (excluding Hong Kong, Macao, Taiwan and the Tibet Autonomous Region) for the period 2007–2019. Among them, green development efficiency is the explanatory variable, which is measured by the superefficiency SBM model considering undesired output. The capital stock in the data used is derived by referring to the calculation method of Shan H J (2008) [36]. ① The depreciation rate is taken as 10.96%. ② Investment price index. Constructed using Shan Haojie’s approach prior to 1994, official published fixed asset price investment index data are used directly after 1994. In particular, for Guangdong, 2001 is used as the cutoff year, as official published fixed asset price investment index data are available only after 2001. ③ Investment in the current year. Replaced by the amount of social fixed asset investment, which is not fundamentally different from gross capital formation. ④ Base year capital stock. The year 1978 is chosen as the base year, and the base year capital stock is the average depreciation rate of 10.96% over 1979 for the amount of society-wide fixed asset investment and the average annual growth rate of investment over the five-year period from 1979 to 1983. Finally, the calculated capital stock data are converted to constant 2007 prices using the investment price index.

CO_2_ emissions are based on the estimation method provided by the UN Intergovernmental Panel on Climate Change. Based on the estimation method provided by the United Nations Intergovernmental Panel on Climate Change, CO_2_ represents estimated carbon dioxide emissions; *i* represents the seven energy sources of coal, coke, paraffin, gasoline, diesel, fuel oil and natural gas; E represents energy consumption; NCV is the average low-level heat content; CEF is the carbon emission factor provided by the IPCC (2006); COF is the carbon oxidation factor; and 44 and 12 are the molecular weights of carbon dioxide and carbon, respectively.

(6) $$CO_{2}=\overset{7}{\underset{i=1}{\sum }}CO_{2,i}=\overset{7}{\underset{i=1}{\sum }}E_{i}\times NCV_{i}\times CEF_{i}\times COF_{i}\times \left(\frac{44}{12}\right)$$

Other economic, social and environmental data are obtained from the China Statistical Yearbook, China Energy Statistical Yearbook, China Environmental Statistical Yearbook, China Science and Technology Statistical Yearbook and provincial statistical yearbooks.

In addition, the paper uses the annual average RMB-to-USD exchange rate. Some economic data denominated in U.S. dollars are converted to RMB. The level of economic development and foreign direct investment are deflated in per capita terms using the GDP per capita deflator with 2007 as the base period. This helps eliminate the effects of inflation and population size. Additionally, the natural logarithm of all data into nonratio form is taken in the empirical analysis. The descriptive statistics of the above variables are shown in Table 2.

## 4. Empirical Analysis

### 4.1. Basic Regression

#### 4.1.1. The Impact of Fiscal Decentralization on Green Development Efficiency

In this paper, model (2) is estimated using the least squares method. Heteroskedasticity-robust standard errors are also used to control the heteroskedasticity problem. Table 3 reports the empirical results from the impact of fiscal decentralization on the efficiency of green development.

(1) The effect of the explanatory variable fiscal decentralization

Model 1 and Model 2 are the estimated results of revenue decentralization. Model 3 and Model 4 are the estimation results of expenditure decentralization. The estimation results of Model 1 and Model 3 show that both revenue decentralization (*FRD1*) and expenditure decentralization (*FED1*) in the linear relationship are negatively related to green development efficiency. The quadratic terms of revenue decentralization (*FRD1*^2^) and expenditure decentralization (*FED1*^2^) are introduced in Model 2 and Model 4, respectively, to examine the nonlinear effects of fiscal decentralization on green development efficiency. The estimated results show that the estimated coefficients of both revenue decentralization and expenditure decentralization are significantly negative, and the estimated coefficients of the quadratic terms are significantly positive. This indicates that both show U-shaped nonlinear characteristics with regard to green development efficiency.

In summary, two conclusions can be drawn from the study. First, fiscal decentralization and green development efficiency in general show a negative relationship during the study period; that is, the current degree of fiscal decentralization in China is not conducive to enhancing the improvement in green development efficiency. Second, the relationship between fiscal decentralization and green development efficiency in China shows a U-shaped characteristic in the long run. When the degree of decentralization is low, the value of green development efficiency decreases as the degree of decentralization increases. The green development efficiency value is promoted only when the degree of decentralization enters a higher stage.

Revenue decentralization measures the financial power held by local government. It reflects the distribution relationship between the central government and local governments in terms of fiscal revenue. When the degree of revenue decentralization is lower, the local government receives less disposable revenue and has relatively limited financial resources. At this time, local governments are more inclined to develop the economy to obtain more economic returns, and in addition, they need to take more responsibility for the supply of public goods and cannot guarantee sufficient financial investment in the field of environmental governance. The elasticity of economic benefits in this period of fiscal decentralization is higher than the elasticity of environmental benefits, and local governments complete the task of economic growth first, while it is difficult for them to consider the supply of public goods, such as environmental governance. As the degree of revenue decentralization increases, local governments with relatively abundant fiscal resources no longer pursue rapid economic growth. Instead, they pay more attention to the high quality of economic growth, take into account the coordinated and sustainable development of the economy and the environment, and allocate more resources to areas with insignificant short-term benefits, such as environmental protection. At this time, the elasticity of the environmental benefits of fiscal decentralization exceeds the elasticity of the economic benefits, and the increase in revenue decentralization is conducive to the improvement in the green development level.

Expenditure decentralization reflects the relationship between the central government and local governments in terms of the allocation of fiscal expenditures. It measures the authority of local government to undertake matters. When the degree of expenditure decentralization is relatively low, the central government assumes more responsibility for the regional public goods supply. Since the environmental pollution status, resource endowment, etc., are not the same across regions, the central government is not aware of the real situation and demand within each region. In the process of allocating financial resources, it tends to ignore the actual preferences for public goods, such as the environment, which in turn inhibits the efficiency of environmental governance. This is not conducive to green development within a region. When the degree of expenditure decentralization is relatively high, local governments assume a greater scope of authority. Local governments have easier access to information on actual demand than the central government and are thus able to supply public goods in line with residents’ real preferences. With the support of information and cost advantages, local governments can better balance the development of economic and environmental harmony. In turn, this can promote the improvement in green development within the region.

(2) Effect of control variables

For the control variables, the estimated coefficient of the level of economic development (*lnPgdp*) is significantly positive. This indicates that as the level of economic development continues to increase, the efficiency of green development also increases. Trade openness (*Ope*) is significantly and negatively correlated with green development efficiency. This indicates that trade openness has a rather negative impact on green development, and when trade openness increases, green development efficiency decreases. Advanced industrial structure (*Ind*) is significantly and positively correlated with green development efficiency. This indicates that the increase in advanced industrial structure is beneficial to the improvement in green development efficiency. The estimated coefficient of foreign direct investment (*lnPfdi*) is positive and fails the significance level test except for Model 2. This indicates that to effectively exert the positive influence of FDI on green development, the structure of introduced foreign investment should be further optimized, and the environmental access threshold should be raised. The estimated coefficient of research and development intensity (*RD*) is positive but not significant. This may be due to insufficient investment in R&D, which has not yet effectively played the role of enhancing the efficiency of regional green development.

#### 4.1.2. Moderating Effect Analysis of Local Government Environmental Preferences

The moderating variables local government environmental preferences *(Pre*) and an interaction term between fiscal decentralization and local government environmental preferences (*FRD1_Pre/FED1_Pre*) were added sequentially in the analysis. To eliminate the effects of multicollinearity among variables, the data were first centered around the construction of the interaction term. The estimated results in Table 4 show that the estimated coefficients of local government environmental preferences are all positive and pass the significance test at the 1% level. This indicates that local government environmental preferences have a significant positive contribution to the improvement in green development efficiency. Taking Model 1 as an example, each 1 percentage point increase in local government environmental preferences increases the value of green development efficiency by 2.981%.

The interaction term between fiscal decentralization and local government environmental preferences is another point of interest in this section. As shown by the estimation results, the signs of the estimated coefficients of the interaction term in Models 2 and 4 are positive, and both are significant at the 1% level. This indicates that the interaction terms positively correlate with the green development efficiency values. The green development efficiency elasticity of the interaction term with the revenue decentralization model is 5.354, and the green development efficiency elasticity of the interaction term with the expenditure decentralization model is 5.468. This implies that there is a significant positive moderating effect of local government environmental preferences on the relationship between fiscal decentralization and green development efficiency. Under the same degree of revenue decentralization or expenditure decentralization, the stronger the environmental preferences of the local government are, the higher the level of green development. It is also found that the primary coefficient of fiscal decentralization is always significantly negative, and the secondary coefficient is always significantly positive. This indicates that there is a stable U-shaped nonlinear relationship between fiscal decentralization and green development efficiency.

### 4.2. Robustness Tests

To prevent changes in the results due to measurement errors and endogeneity, this paper uses three means of robustness analysis: alternative core variables, alternative estimation methods and the instrumental variables method. The aim is to enhance the credibility and scientific validity of the above findings.

#### 4.2.1. Endogeneity Analysis

We next address the potential endogeneity problem in the empirical model owing to two-way causality between fiscal decentralization and the green development level. We discuss this using the instrumental variables approach. The weighted average of individual fiscal decentralization (the weight is the inverse of the geographical distance between regions) and the lagged term of fiscal decentralization are used as the instrumental variables of fiscal decentralization in a region. Two-stage least squares (2SLS) estimation was chosen to overcome the estimation bias due to the aforementioned reciprocal causality and the possible omitted variable problem. The model regression results are reported in Table 5.

First, the Kleibergen–Paap rk LM statistics are all highly significant. This indicates that there is no under-identification of instrumental variables problem. The Cragg-Donald Wald F statistics are all greater than the Stock-Yogo weak identification test critical value at the 10% level, which indicates that there is no weak instrumental variable problem. The Sargan test results show that there is no overidentification problem and that the instrumental variables satisfy the exogenous rule. The above results indicate that the selected instrumental variables are valid. From the 2SLS estimation results, we know that the estimated coefficient of the primary term of fiscal decentralization is significantly negative, the coefficient of the secondary term is significantly positive, and the estimated coefficient of the interaction term of fiscal decentralization and local government environmental preferences is significantly positive. All these results are consistent with the baseline regression results, and none of the coefficients change significantly in absolute magnitude. This indicates that the findings of the benchmark regression are still robust after considering the exogeneity issue.

#### 4.2.2. Substitution Variables Test

The second set of core variables is substituted into model (3). As shown by the estimation results (Table 6), although there is some underestimation of the effect of the proxy variables on green economic efficiency, the sign and significance level of the regression coefficients remain consistent with the baseline regression results. This indicates that the estimated results do not change significantly due to changes in the measures of the core explanatory variables. The findings of the study are robust in general.

#### 4.2.3. Replacement Model Test

From the estimation results (Table 7), it can be seen that fiscal decentralization (*FRD1, FED1*) continues to have a U-shaped relationship with green development efficiency. The estimated coefficients of local government environmental preferences (*Pre*), the estimated coefficients of the interaction term between fiscal decentralization and local government environmental preferences (*FRD1_Pre, FED1_Pre*), and the estimated coefficients of the spatial lagged regression coefficient (ρ) are all significantly positive. All of them pass the 1% significance level test.

The sign and significance level of the core variables remain consistent with the benchmark regression results after taking into account the green development efficiency spillover effects between regions. This further verifies the robustness of the baseline regression results. The study also finds that the green development efficiency of a region is influenced by its own factors and the green development efficiency of other neighboring regions. The improvement in green development efficiency in neighboring regions has a demonstration effect and a driving effect on the local region. It can induce local governments to adjust their environmental preferences and engage in “top-to-top competition” for green development.

### 4.3. Heterogeneity Analysis

The behavioral preferences of local government are strongly related to local resource endowments. Regional differences should be fully considered when exploring the relationship between fiscal decentralization, local government environmental preferences, and green development. Therefore, the study divides the national sample into coastal and inland regions and sets the regional dummy variable *dum* (if the region belongs to the coastal region, *dum* = 0, and vice versa *dum* = 1). Table 8 reports the model estimation results. The sign and significance level of the estimated coefficients of the core variables remain consistent with the full sample regression results of coastal and inland regions. This indicates that local government environmental preferences moderate the effect of fiscal decentralization on green development efficiency.

The estimated coefficients on the interaction term with coastal areas are 8.465 (*FRD1_Pre*) and 8.782 (*FED1_Pre*). The estimated coefficients for inland areas are 4.309 and 4.646. This implies that the moderating effect of local government environmental preferences is greater in coastal areas. This is related to local government dynamism and influenced by economic power. The coastal areas are at a relatively high stage of development. In addition, their stronger economies make them better equipped to invest more resources in environmental protection and to mitigate the contradictory relationship between economic growth and environmental protection. Therefore, local government environmental preferences can ease the inhibiting effect of fiscal decentralization on the efficiency of green development to a greater extent.

### 4.4. Further Discussion

The above paper has confirmed the moderating effect of local government environmental preferences on the relationship between fiscal decentralization and green development efficiency. To further investigate the variability of the effect of fiscal decentralization on green development efficiency under different local government environmental preferences, we set local government environmental preferences as the threshold variable for threshold effect analysis.

#### 4.4.1. Threshold Effect Test of Local Government Environmental Preferences

The results of the threshold effect test show that regardless of whether the explanatory variable affected by the threshold variable local government environmental preferences is revenue decentralization or expenditure decentralization, all pass the single threshold test, and none pass the double or triple threshold tests (Table 9). The estimation results of the single-threshold model show that the effects of both revenue decentralization and expenditure decentralization on green development efficiency change structurally when the environmental preferences of local government cross 0.0234.

**Figure 1:**
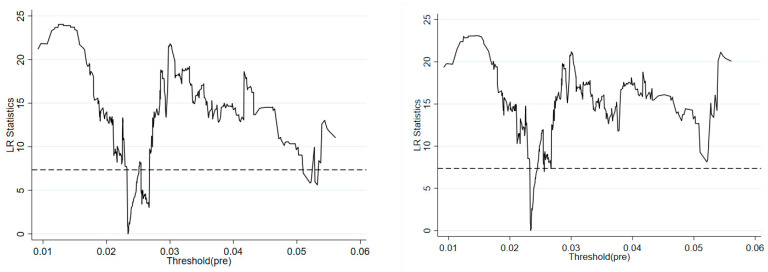
Likelihood ratio function diagram.

To more clearly observe the process of constructing the threshold estimates and confidence intervals and to test the validity of the thresholds, Figure 1 reports the likelihood ratio function plots (the variable influenced by the threshold variable local government environmental preferences in the left panel is revenue decentralization, while that in the right panel is expenditure decentralization). It can be clearly seen that the single threshold effects are both significant.

#### 4.4.2. Threshold Model Regression Results Analysis

From the estimation results of Model 1, we can see that the sign of the estimated coefficient of revenue decentralization is positive when the environmental preferences of local government are below the threshold value of 0.0234, and the results pass the 1% significance level test. This indicates that revenue decentralization significantly inhibits the improvement in green development efficiency. When preferences are higher than the threshold value of 0.0234, the estimated coefficient of revenue decentralization has a negative sign but is not significant. This indicates that revenue decentralization does not show a significant negative effect on green development efficiency. The estimation results of Model 2 show that expenditure decentralization significantly inhibits green development efficiency when local government environmental preferences are below the threshold value of 0.0234; when local government environmental preferences are above the threshold value of 0.0234, expenditure decentralization is still significantly and negatively related to green development efficiency. However, the absolute value of the estimated coefficient decreases from 0.218 to 0.150, which indicates that the negative effect of expenditure decentralization on green development efficiency is significantly reduced (Table 10). In summary, it can be seen that the negative effect of fiscal decentralization on green development efficiency is mitigated as the environmental preferences of local governments increase. This further validates the baseline regression results.

## 5. Conclusions

In the context of the comprehensive promotion of green development, the study constructs an analytical framework that includes fiscal decentralization, local government environmental preferences, and green development. Green development efficiency, measured by the superefficiency SBM model including undesired output, is taken as the explanatory variable. The two-way fixed effects model and panel threshold model are used to empirically test the effect of fiscal decentralization on green development under the regulation of local government environmental preferences. The following main research findings are obtained.

First, both revenue decentralization and expenditure decentralization have a U-shaped nonlinear relationship with green development efficiency. That is, a lower level of fiscal decentralization is not conducive to improving green development efficiency. It promotes the improvement in green development efficiency only when decentralization is at a high level. At this stage, fiscal decentralization in China has not yet had a positive effect on green development efficiency.

Second, local government environmental preferences are positively related to green development efficiency. Regardless of revenue decentralization or expenditure decentralization, local government environmental preferences have a positive moderating effect on the relationship between fiscal decentralization and green development efficiency. Under the same degree of fiscal decentralization, the stronger the environmental preferences of the local government are, the higher the green development efficiency. In addition, the subregional results show that the moderating effect of local government environmental preferences is greater in coastal areas than inland areas.

Third, the effect of fiscal decentralization on green development varies based on different strengths of government environmental preferences. When local government environmental preferences are below the threshold, fiscal decentralization significantly inhibits the improvement in green development efficiency. The negative effect decreases significantly after the threshold is crossed; that is, the negative effect of fiscal decentralization on green development efficiency is mitigated as the environmental preferences of local governments increase.

## 6. Policy Recommendations

First, the government performance appraisal system should be optimized, and the green GDP performance appraisal mechanism should be improved. When assessing the government’s performance, the proportion of green development indicators should be appropriately increased. The binding indicators for energy conservation and emission reduction should be clarified, and environmental governance should meet the standards. The results of green performance appraisal should be taken as an important basis of officials’ promotion to strengthen local government awareness of green development. A corresponding accountability mechanism should be established and improved. The subjects responsible for environmental problems and the boundaries of responsibility should be clarified. The legal responsibility of relevant personnel should be strictly pursued, and a lifelong accountability system for environmental protection failure should be implemented. The supervisory role of public and public opinion in green performance assessment should be effectively undertaken, incorporating public opinion about assessment and decisively combating phenomena such as superficial studies. The objectivity and fairness of the assessment results should be guaranteed so that assessment findings are put into practice.

Second, the reform of fiscal decentralization should be deepened. We should accelerate the improvement in the division of fiscal power between the central government and local governments. Based on the principle of the relative equality of financial and administrative powers, local governments should be given a greater degree of financial autonomy in decision making to make the effect of fiscal decentralization on the efficiency of green development cross the inflection point as soon as possible. The environmental protection transfer payment system should be further standardized. After fully considering the actual situation in terms of regional economic development levels and based on the principle of local conditions, the environmental protection transfer payments to each locality should be strengthened. The supervision of transfer payments, the information disclosure system of environmental protection transfer payments, and regulation of the use of funds should also be strengthened to ensure that the transfer payments are truly used in the field of environmental protection [37,38].

Third, local financial investment in the field of environmental protection should be increased, taking environmental protection investment as a key element of local fiscal expenditures. We should optimize the structure of fiscal expenditures and increase the absolute and relative scale of fiscal environmental protection expenditures. In addition, we should gradually establish a long-term growth mechanism to effectively support the irreplaceable role of environmental protection spending in enhancing the level of regional green development. We should deepen the vertical management reform of environmental protection agencies, further increase the relative independence of environmental protection departments, and reduce interference in financial, material and human resource input in the environmental protection field. We should guarantee that the financial funds for environmental protection can be effectively used for energy conservation, emission reduction, and environmental governance, give full play to the leverage of financial funds, and guide more social capital to enter the environmental protection field through tax relief, government subsidies and other preferential policies. Moreover, we should increase the attention of all social strata to the environmental protection industry and help each region achieve its green development goals.

Fourth, we should promote synergistic regional development. Full play should be given to the positive spatial spillover effect of green development efficiency. We should break down barriers to the flow of information and factors and promote the reasonable flow of capital and technology and other factors between regions. Regions with lower green development levels can promote their own green development levels by learning and absorbing relevant successful experience and introducing advanced production technologies and finally realizing the overall improvement in the green development level. We should deepen and promote the joint prevention and treatment of the environment, overcome the limitations of administrative boundaries, and unify the relevant environmental pollution emission standards of neighboring or nearby regions. Environmental monitoring data can be shared by forming regional monitoring networks. We should conduct regular joint enforcement inspections and enhance the traceability of cross-border pollution, jointly cracking down on environmental violations to prevent the free-rider mentality.

The research object of the article is limited to the provincial level. It fails to examine in depth the impact of fiscal decentralization on green development and the moderating effect of local government environmental preferences on the relationship between the two at the city level. The heterogeneity analysis is also not rich enough due to the limited number of samples at the provincial level. This weakens the persuasive power of the article’s conclusions to some extent.

## Figures and Tables

**Table 1:** Spatial weight matrix setting.

	*In_1_*		*In_2_*		*In_3_*	
*w*_ij_	1	0	1/d_ij_	0	1/|avgPgdp_i_-avgPgdp_j_|	0
	i ≠ j and there is a common boundary	Others	i ≠ j, d_ij_ is the latitude and longitude surface distance of the provincial capital city	i = j	i ≠ j, AVGpgdp is the average GDP per capita of the sample during the study period	i = j

**Table 2:** Descriptive statistics for each variable.

*Indicators*	*Symbol*	*Unit*	*Number of Observations*	*Average Value*	*Standard Deviation*	*Minimum Value*	*Maximum Value*
*Capital Stock*	*CS*	RMB 100 million	390	54,559.61	47,546.3	2161.86	263,000
*Year-End Employed Persons*	*YEP*	10,000 persons	390	2672.167	1759.102	298.56	7150.25
*Total Energy Consumption*	*TES*	10,000 tons of sce	390	13,970.2	8585.293	1057	41,390
*Area of Urban Districts*	*AUD*	sq·km	390	1588.846	1158.539	110.65	6397.7
*Gross Domestic Product*	*GDP*	RMB 100 million	390	17,245.64	14,963.06	720.1	85,150.93
*Volume of CO_2_ Discharged*	*CO_2_*	10,000 tons	390	35,868.68	25,978.01	2089.607	118,000
*(Volume of SO_2_ Discharged*	*SO_2_*	10,000 tons	390	55.991	42.835	0.192	182.74
*Volume of COD Discharged*	*COD*	10,000 tons	390	49.75	42.141	1.968	198.25
*Volume of Industrial Solid Waste Produced*	*ISWP*	10,000 tons	390	10,359.23	9316.851	158	52,037
*Green Development Efficiency*	*GDE*	%	390	0.387	0.251	0.199	1.178
*Fiscal Revenue Decentralization*	*FRD1*	%	390	0.582	0.462	0.193	2.588
*Fiscal Expenditure Decentralization*	*FED1*	%	390	0.992	0.435	0.513	2.806
*Local Government Environmental Preferences*	*Pre*	%	390	0.031	0.011	0.008	0.068
*Per Capita Gdp*	*lnPgdp*	RMB	390	10.43	0.556	8.841	11.801
*Trade Openness Degree*	*Ope*	%	390	0.291	0.312	0.011	1.594
*Advanced Industrial Structure*	*Ind*	%	390	1.204	0.674	0.527	5.234
*Per Capita Foreign Direct Investment*	*lnPfdi*	RMB	390	6.186	1.354	1.413	8.987
*Research And Development Intensity*	*RD*	%	390	0.016	0.011	0.002	0.062

**Table 3:** Regression results of the effect of fiscal decentralization on green development efficiency.

	*Model 1*	*Model 2*	*Model 3*	*Model 4*
*Explained Variables*	*GDE*	*GDE*	*GDE*	*GDE*
*FRD1*	−0.230	−0.550 *		
	(−1.72)	(−2.03)		
*FRD1^2^*		0.106 **		
		(2.40)		
*FED1*			−0.199 **	−0.451 ***
			(−2.74)	(−3.58)
*FED1^2^*				0.0709 **
				(2.89)
*lnPgdp*	0.242 *	0.294 *	0.247 **	0.292 **
	(1.82)	(1.83)	(2.26)	(2.49)
*Ope*	−0.340 **	−0.340 **	−0.310 *	−0.312 *
	(−2.29)	(−2.33)	(−1.85)	(−1.81)
*Ind*	0.137 ***	0.141 ***	0.131 **	0.141 ***
	(3.67)	(3.62)	(2.99)	(3.15)
*lnPfdi*	0.00867	0.0130*	0.00420	0.00715
	(1.48)	(2.14)	(0.40)	(0.69)
*RD*	6.486	7.033	5.334	5.648
	(0.95)	(1.00)	(0.84)	(0.89)
*_cons*	−2.007	−2.439	−1.955 *	−2.284 *
	(−1.50)	(−1.54)	(−1.87)	(−2.06)
Province fixed effect	YES	YES	YES	YES
Year-fixed effect	YES	YES	YES	YES
*N*	390	390	390	390
*R^2^*	0.357	0.365	0.363	0.367
*F*	92.24 ***	102.36 ***	80.41 ***	78.82 ***

Notes: t-statistics are in parentheses. ***, **, and * indicate significance at the 1%, 5%, and 10% levels, respectively.

**Table 4:** Regression results of the moderating effect of local government environmental preferences.

	*Model 1*	*Model 2*	*Model 3*	*Model 4*
*Explained Variables*	*GDE*	*GDE*	*GDE*	*GDE*
*FRD1*	−0.558 **	−0.640 **		
	(−2.42)	(−2.48)		
*FRD1^2^*	0.109 ***	0.154 ***		
	(3.45)	(3.21)		
*FED1*			−0.421 ***	−0.543 ***
			(−4.23)	(−3.73)
*FED1^2^*			0.0646 **	0.108 ***
			(3.01)	(3.08)
*Pre*	2.984 ***	2.047 ***	2.817 ***	1.901 ***
	(8.67)	(5.78)	(6.64)	(7.46)
*FRD1_Pre*		5.354 ***		
		(9.21)		
*FED1_Pre*				5.468 ***
				(5.22)
*Pgdp*	0.380 **	0.440 **	0.359 ***	0.396 ***
	(2.46)	(2.87)	(3.37)	(3.56)
*Ope*	−0.310 **	−0.267 **	−0.283	−0.261 *
	(−2.33)	(−2.42)	(−1.76)	(−1.83)
*Ind*	0.118 **	0.0632	0.117 **	0.0555
	(3.05)	(1.41)	(2.70)	(1.33)
*Pfdi*	0.00987	0.00946	0.00380	0.00237
	(1.19)	(1.19)	(0.34)	(0.22)
*RD*	5.568	7.823	4.232	6.206
	(0.90)	(1.59)	(0.75)	(1.31)
*_cons*	−3.324 **	−3.845 **	−2.992 **	−3.221 **
	(−2.18)	(−2.56)	(−2.96)	(−3.05)
Province fixed effect	YES	YES	YES	YES
Year-fixed effect	YES	YES	YES	YES
*N*	390	390	390	390
*R^2^*	0.390	0.423	0.390	0.418
*F*	140.69 ***	311.70 ***	300.38 ***	177.87 ***

Notes: t-statistics are in parentheses. ***, **, and * indicate significance at the 1%, 5%, and 10% levels, respectively.

**Table 5:** Regression results after considering the exogeneity problem.

	*Model 1*	*Model 2*	*Model 3*	*Model 4*	*Model 5*	*Model 6*
*Explained Variables*	*GDE*	*GDE*	*GDE*	*GDE*	*GDE*	*GDE*
*FRD1*	−0.834 **	−0.782 **	−0.842 ***			
	(−2.44)	(−2.55)	(−3.00)			
*FRD1^2^*	0.170 **	0.162 **	0.214 ***			
	(2.40)	(2.44)	(3.31)			
*FED1*				−0.872 ***	−0.788 ***	−0.960 ***
				(−2.66)	(−2.62)	(−3.32)
*FED1^2^*				0.155 **	0.139 **	0.202 ***
				(2.30)	(2.20)	(3.11)
*Pre*		2.997 **	2.045 **		2.717 **	1.783 *
		(2.53)	(2.18)		(2.36)	(1.89)
*FRD1_Pre*			5.444 ***			
			(4.44)			
*FED1_Pre*						5.569 ***
						(3.30)
Control variables	YES	YES	YES	YES	YES	YES
Province fixed effect	YES	YES	YES	YES	YES	YES
Year-fixed effect	YES	YES	YES	YES	YES	YES
*N*	390	390	390	390	390	390
*R^2^*	0.359	0.387	0.420	0.354	0.379	0.407
*F*	8.23 ***	8.25 ***	9.91 ***	8.30 ***	8.43 ***	10.47 ***
*Kleibergen-Paap rk LM*	72.658 ***	71.915 ***	75.072 ***	57.798 ***	58.892 ***	58.442 ***
*Cragg-Donald Wald F*	97.314	98.803	70.402	95.783	95.463	65.152
*Sargan-p*	0.1122	0.2872	0.3092	0.2903	0.3873	0.4125

Notes: t-statistics are in parentheses. ***, **, and * indicate significance at the 1%, 5%, and 10% levels, respectively.

**Table 6:** Regression results for replacing indicators of explanatory variables.

	*Model 1*	*Model 2*	*Model 3*	*Model 4*	*Model 5*	*Model 6*
*Explained Variables*	*GDE*	*GDE*	*GDE*	*GDE*	*GDE*	*GDE*
*FRD2*	−0.303 *	−0.306 *	−0.297 *			
	(−1.91)	(−2.16)	(−1.97)			
*FRD2^2^*	0.0380 *	0.0375 **	0.0346 *			
	(2.13)	(2.44)	(1.90)			
*FED2*				−0.132 ***	−0.122 **	−0.0995 **
				(−3.37)	(−3.03)	(−2.94)
*FED2^2^*				0.00466 ***	0.00428 **	0.00272 *
				(3.08)	(2.61)	(2.07)
*Pre*		3.008 ***	2.172 ***		2.513 ***	1.915 ***
		(8.24)	(5.51)		(4.89)	(6.41)
*FRD2_Pre*			2.234 ***			
			(7.98)			
*FED2_Pre*						0.689 ***
						(6.91)
*Control variables*	Control	Control	Control	Control	Control	Control
*_cons*	−1.985	−2.955 **	−3.656 **	−3.536 **	−3.991 ***	−3.675 **
	(−1.42)	(−2.27)	(−2.53)	(−2.64)	(−3.22)	(−3.01)
Province fixed effect	YES	YES	YES	YES	YES	YES
Year-fixed effect	YES	YES	YES	YES	YES	YES
*N*	390	390	390	390	390	390
*R^2^*	0.364	0.390	0.418	0.390	0.407	0.427
*F*	109.60 ***	137.94 ***	146.60 ***	105.34 ***	224.02 ***	265.57 ***

Notes: t-statistics are in parentheses. ***, **, and * indicate significance at the 1%, 5%, and 10% levels, respectively.

**Table 7:** Spatial lag model regression results.

	*Model 1*	*Model 2*	*Model 3*	*Model 4*	*Model 5*	*Model 6*
*Spatial Weight Matrix*	*In_1_*		*In_2_*		*In_3_*	
*Explained Variables*	*GDE*	*GDE*	*GDE*	*GDE*	*GDE*	*GDE*
*FRD1*	−0.627 ***		−0.632 ***		−0.588 ***	
	(−4.17)		(−4.06)		(−3.79)	
*FRD1^2^*	0.156 ***		0.153 ***		0.141 ***	
	(3.48)		(3.30)		(3.06)	
*FED1*		−0.544 ***		−0.530 ***		−0.505 ***
		(−3.40)		(−3.22)		(−3.09)
*FED1^2^*		0.114 ***		0.106 **		0.0999 **
		(2.63)		(2.38)		(2.27)
*Pre*	1.601 **	1.505 **	1.803 **	1.711 **	1.795 **	1.630 **
	(2.20)	(2.04)	(2.39)	(2.24)	(2.41)	(2.17)
*FRD1_Pre*	5.361 ***		5.485 ***		5.029 ***	
	(4.94)		(4.89)		(4.50)	
*FED1_Pre*		5.339 ***		5.531 ***		5.145 ***
		(4.37)		(4.40)		(4.13)
*Control variables*	Control	Control	Control	Control	Control	Control
R	0.316 ***	0.301 ***	0.301 **	0.252 *	0.216 ***	0.236 ***
	(4.87)	(4.57)	(2.07)	(1.67)	(2.67)	(2.93)
Log-likelihood	415.845	412.941	407.228	404.909	408.823	407.586
Province fixed effect	YES	YES	YES	YES	YES	YES
Year-fixed effect	YES	YES	YES	YES	YES	YES
*N*	390	390	390	390	390	390
*R^2^*	0.154	0.159	0.145	0.152	0.158	0.159

Notes: t-statistics are in parentheses. ***, **, and * indicate significance at the 1%, 5%, and 10% levels, respectively.

**Table 8:** Subregional regression results.

	*Model 1*	*Model 2*
*Explained Variables*	*GDE*	*GDE*
*FRD1*	−0.655 *	
	(−2.14)	
*FRD1^2^*	0.165 **	
	(2.43)	
*FED1*		−0.654 ***
		(−3.16)
*FED1^2^*		0.154 ***
		(4.12)
*Pre*	2.351 **	3.617 ***
	(2.52)	(4.14)
*FRD1_Pre*	8.465 ***	
	(7.68)	
*FED1_Pre*		8.782 ***
		(10.64)
dum * FRD1	0.0803	
	(0.36)	
dum * FRD1^2^	−0.00632	
	(−0.11)	
dum * FED1		0.250
		(1.12)
dum * FED1^2^		−0.0851
		(−1.70)
dum * Pre	−0.750	−2.695
	(−0.39)	(−1.58)
dum * FRD1_Pre	−4.156 ***	
	(−3.92)	
dum * FED1_Pre		−4.136 ***
		(−3.42)
*Control variables*	Control	Control
*_cons*	−3.391 **	−2.844 **
	(−2.37)	(−2.75)
Province fixed effect	YES	YES
Year-fixed effect	YES	YES
*N*	390	390
*R^2^*	0.433	0.430
*F*	5596.83 ***	1084.12 ***

Notes: t-statistics are in parentheses. ***, **, and * indicate significance at the 1%, 5%, and 10% levels, respectively.

**Table 9:** Results of the threshold effect test.

Threshold Variables	Variables Affected by Threshold Variables	Threshold Type	F-Statistic	*p*-Value	Threshold			Estimated Value
					10%	5%	1%	
*Local government environmental preferences (Pre)*	*Revenue decentralization (FRD1)*	Single Threshold	25.32	0.060	19.9383	26.1949	48.2173	0.0234
		Double Threshold	12.02	0.222	16.6971	22.0723	35.8549	
		Threefold threshold	4.59	0.818	21.0326	26.2724	42.9031	
	*Expenditure decentralization (FED1)*	Single Threshold	24.31	0.048	19.3646	23.7335	44.3676	0.0234
		Double Threshold	11.50	0.292	18.0160	21.3780	36.8583	
		Threefold threshold	12.36	0.136	13.6849	17.2283	26.0970	

Note: *p*-statistics and critical statistics are the results obtained by repeated sampling 500 times using the self-sampling method.

**Table 10:** Threshold model regression results.

	*Model 1*	*Model 2*
*Explained Variables*	*GDE*	*GDE*
*FRD1 (Pre ≤ 0.0234)*	−0.222 ***	
	(−2.80)	
*FRD1 (Pre > 0.0234)*	−0.131	
	(−1.60)	
*FED1 (Pre ≤ 0.0234)*		−0.218 ***
		(−3.83)
*FED1 (Pre > 0.0234)*		−0.150 ***
		(−2.59)
*Control variables*	Control	Control
*_cons*	−2.483 *	−2.423 *
	(−1.75)	(−1.81)
Province fixed effect	YES	YES
Year-fixed effect	YES	YES
*N*	390	390
*R^2^*	0.396	0.401
*F*	11.76 ***	12.01 ***

Notes: t-statistics are in parentheses. *** and * indicate significance at the 1% and 10% levels, respectively.

## Data Availability

Not applicable.
